# Supplementary material for: Human norovirus (HuNoV) GII RNA in wastewater solids at 145 United States wastewater treatment plants: comparison to positivity rates of clinical specimens and modeled estimates of HuNoV GII shedders
Source: J Expo Sci Environ Epidemiol. 2023 Aug 7;34(3):440–7. doi: 10.1038/s41370-023-00592-4 (PMC11222142; doi:10.1038/s41370-023-00592-4)
Supplement: Supplementary file 1 — Reporting Checklist [file 41370_2023_592_MOESM1_ESM.pdf]

SPRINGER NATURE

Corresponding Author name: Boehm

Manuscript Number: JESEE-23-4301.R1

Reporting Checklist

This checklist is used to ensure the quality, transparency, and reproducibility of published results. We require authors attest that these components have been considered and addressed.

**Exposure Assessment Guiding Principle**	**Yes/No/Not Applicable**
Has the method to estimate exposure been described clearly?	Not applicable
Has the exposure assessment method been validated/evaluated as a proxy for exposure and is its validity or agreement with other methods described?	Not applicable
Is the time period over which the exposure assessment method is considered to be a proxy for exposure appropriate for the research question?	Not applicable
If exposure is modeled or measured, were all critical potential routes and sources of exposure considered?	Not applicable
If exposure is modeled, how does it vary over space and time and are necessary historical data incorporated?	Not applicable
If biomarkers are used as indicators of exposure, could the biomarker measurement have been affected by the outcome (i.e., reverse causality)?	Not applicable
Are the strengths and weaknesses of the exposure approach detailed and discussed?	Not applicable
