# Supplementary material for: Human norovirus (HuNoV) GII RNA in wastewater solids at 145 United States wastewater treatment plants: comparison to positivity rates of clinical specimens and modeled estimates of HuNoV GII shedders
Source: J Expo Sci Environ Epidemiol. 2023 Aug 7;34(3):440–7. doi: 10.1038/s41370-023-00592-4 (PMC11222142; doi:10.1038/s41370-023-00592-4)
Supplement: Supplementary file 2 — Supplementary Information [file 41370_2023_592_MOESM2_ESM.pdf]

Supporting Information
for
Human norovirus (HuNoV) GII RNA in wastewater solids at 145 United States wastewater treatment plants: Comparison to positivity rates of clinical specimens and modeled estimates of HuNoV GII shedders

Authors
Alexandria B. Boehm[1*], Marlene K. Wolfe[2], Bradley White[3], Bridgette Hughes[3], Dorothea Duong[3], Niaz Banaei[4], Amanda Bidwell[1]

Affiliations
1. Department of Civil & Environmental Engineering, School of Engineering and Doerr School of Sustainability, Stanford University, Stanford, CA, USA
2. Gangarosa Department of Environmental Health, Rollins School of Public Health, Emory University, Atlanta, GA, USA
3. Verily Life Sciences, South San Francisco, CA, USA
4. Stanford Health Care Clinical Microbiology Laboratory, Stanford University School of Medicine, Palo Alto, CA, USA

*corresponding author: Alexandria Boehm (aboehm@stanford.edu)

Calculation of population weighted average HuNoV and HuNoV/PMMoV

The population weighted average HuNoV and HuNoV/PMMoV is calculated using the raw data for each site. For each date, and individual WWTP, the value measured for the day is multiplied by the WWTP's population served, as reported by the WWTP. These products are then summed across each day, and divided by the total population of all WWTPs. This calculation results in the population weighted average for day d, as in the following equation for the 145 selected plants:

$$Population\ weighted\ average\ of\ Z(d)\ = \left(\sum_{n=1}^{145} pop_n * Z(d)\right) / \left(\sum_{n=1}^{145} pop_n\right)$$

Where Z(d) is either HuNoV or HuNoV/PMMoV on day d, pop_n is the population served by WWTP n,and n is a counter that goes from 1 to 145 and covers each WWTP. Prior to calculating the aggregated line, non-detect values are set to 500 cp/g (approximately the limit of detection for HuNoV), and for Z = HuNoV/PMMoV, non-detects were set to 500 cp/g normalized by the average PMMoV value for the WWTP. For days when a WWTP did not have a sample, Z was estimated using linear interpolation between the previous and next values with respect to d.

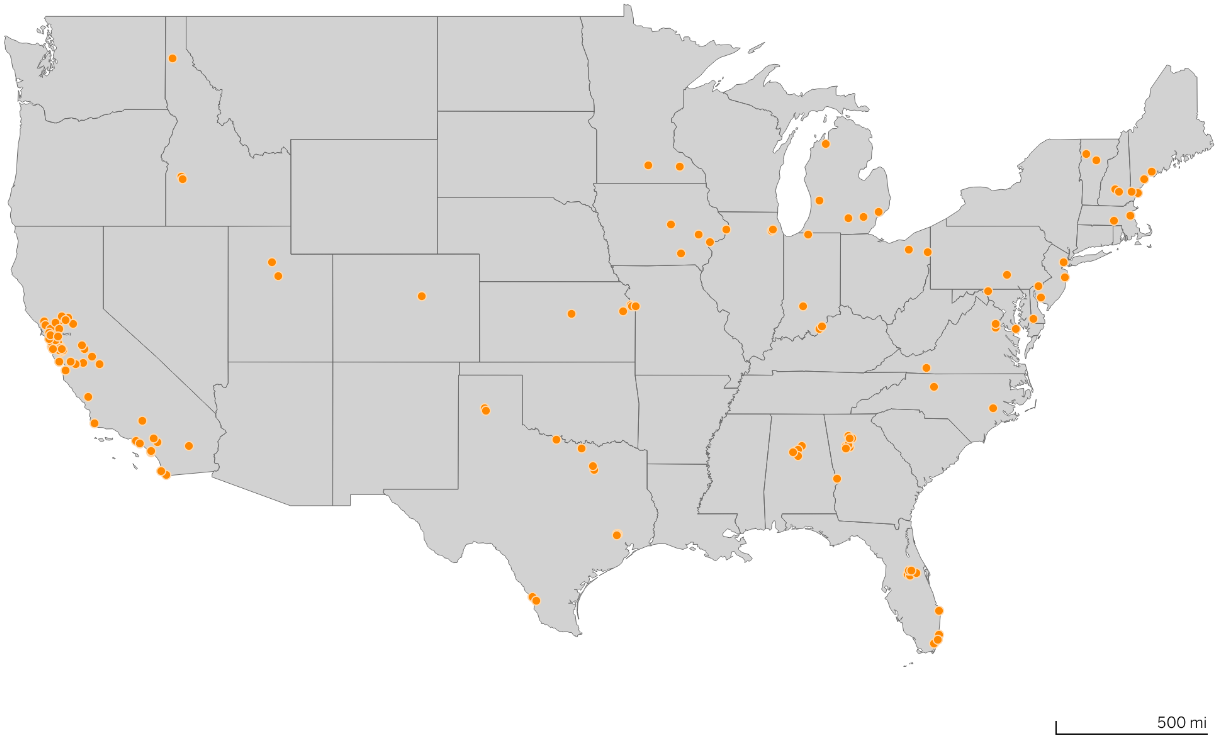

Figure S1. Map of 145 WWTPs. Orange dot indicates WWTP location.

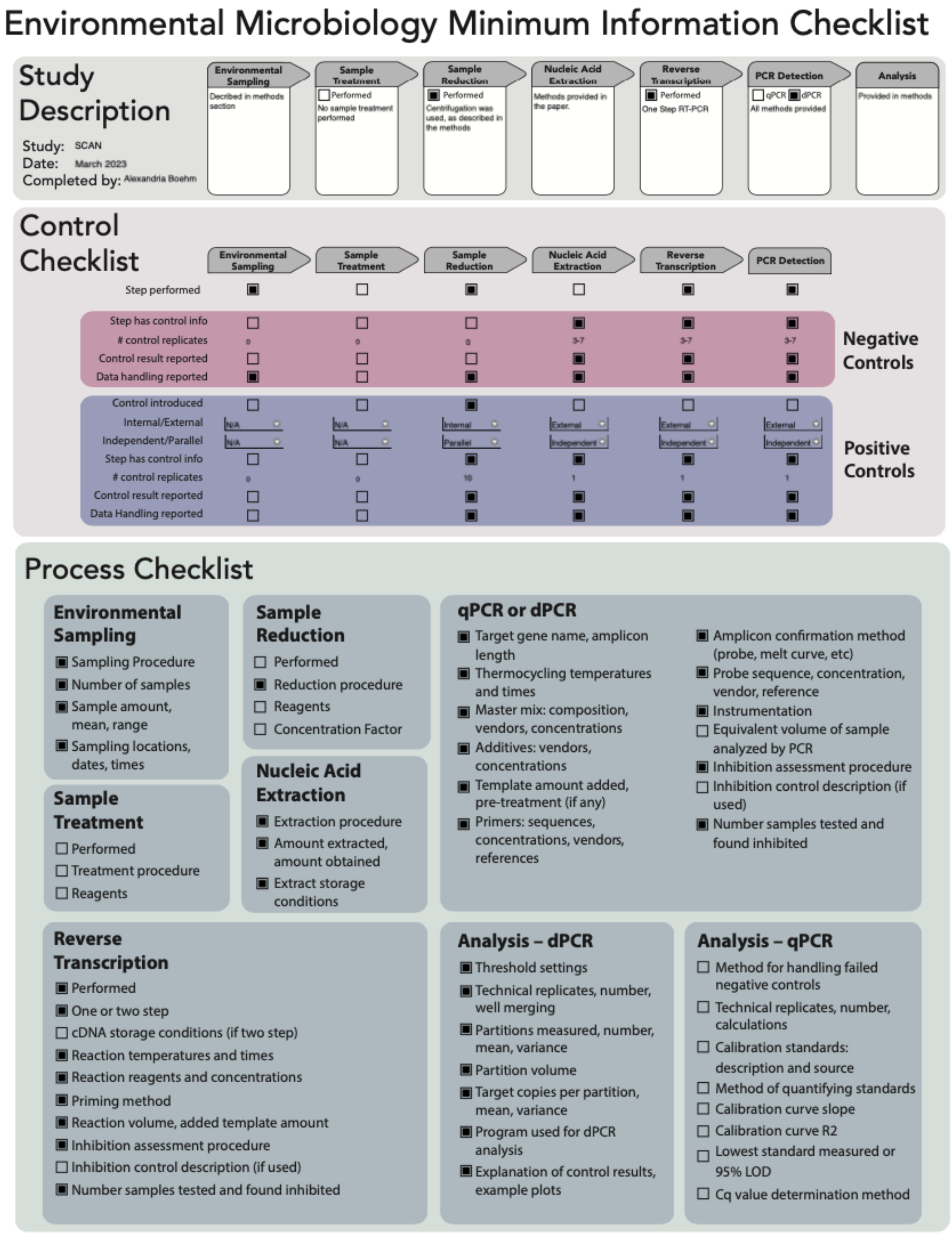

Environmental Microbiology Minimum Information Checklist

Study Description

Study: SCAN
Date: March 2023
Completed by: Alexandria Boehm

Environmental Sampling	Sample Treatment	Sample Reduction	Nucleic Acid Extraction	Reverse Transcription	PCR Detection	Analysis
Decribed in methods section	☐ Performed<br>No sample treatment performed	■ Performed<br>Centrifugation was used, as described in the methods	Methods provided in the paper.	■ Performed<br>One Step RT-PCR	☐ qPCR ■ dPCR<br>All methods provided	Provided in methods

Control Checklist

	Environmental Sampling	Sample Treatment	Sample Reduction	Nucleic Acid Extraction	Reverse Transcription	PCR Detection
Step performed	■	☐	■	☐	■	■
**Negative Controls**						
Step has control info	☐	☐	☐	■	■	■
# control replicates	0	0	0	3-7	3-7	3-7
Control result reported	☐	☐	☐	■	■	■
Data handling reported	■	☐	■	■	■	■
**Positive Controls**						
Control introduced	☐	☐	■	☐	☐	☐
Internal/External	N/A	N/A	Internal	External	External	External
Independent/Parallel	N/A	N/A	Parallel	Independent	Independent	Independent
Step has control info	☐	☐	■	■	■	■
# control replicates	0	0	10	1	1	1
Control result reported	☐	☐	■	■	■	■
Data Handling reported	☐	☐	■	■	■	■

Process Checklist

Environmental Sampling
- ■ Sampling Procedure
- ■ Number of samples
- ■ Sample amount, mean, range
- ■ Sampling locations, dates, times

Sample Treatment
- ☐ Performed
- ☐ Treatment procedure
- ☐ Reagents

Sample Reduction
- ☐ Performed
- ■ Reduction procedure
- ☐ Reagents
- ☐ Concentration Factor

Nucleic Acid Extraction
- ■ Extraction procedure
- ■ Amount extracted, amount obtained
- ■ Extract storage conditions

qPCR or dPCR
- ■ Target gene name, amplicon length
- ■ Thermocycling temperatures and times
- ■ Master mix: composition, vendors, concentrations
- ■ Additives: vendors, concentrations
- ■ Template amount added, pre-treatment (if any)
- ■ Primers: sequences, concentrations, vendors, references
- ■ Amplicon confirmation method (probe, melt curve, etc)
- ■ Probe sequence, concentration, vendor, reference
- ■ Instrumentation
- ☐ Equivalent volume of sample analyzed by PCR
- ■ Inhibition assessment procedure
- ☐ Inhibition control description (if used)
- ■ Number samples tested and found inhibited

Reverse Transcription
- ■ Performed
- ■ One or two step
- ☐ cDNA storage conditions (if two step)
- ■ Reaction temperatures and times
- ■ Reaction reagents and concentrations
- ■ Priming method
- ■ Reaction volume, added template amount
- ■ Inhibition assessment procedure
- ☐ Inhibition control description (if used)
- ■ Number samples tested and found inhibited

Analysis – dPCR
- ■ Threshold settings
- ■ Technical replicates, number, well merging
- ■ Partitions measured, number, mean, variance
- ■ Partition volume
- ■ Target copies per partition, mean, variance
- ■ Program used for dPCR analysis
- ■ Explanation of control results, example plots

Analysis – qPCR
- ☐ Method for handling failed negative controls
- ☐ Technical replicates, number, calculations
- ☐ Calibration standards: description and source
- ☐ Method of quantifying standards
- ☐ Calibration curve slope
- ☐ Calibration curve R2
- ☐ Lowest standard measured or 95% LOD
- ☐ Cq value determination method

Figure S2. EMMI checklist from Borchardt et al.[1]

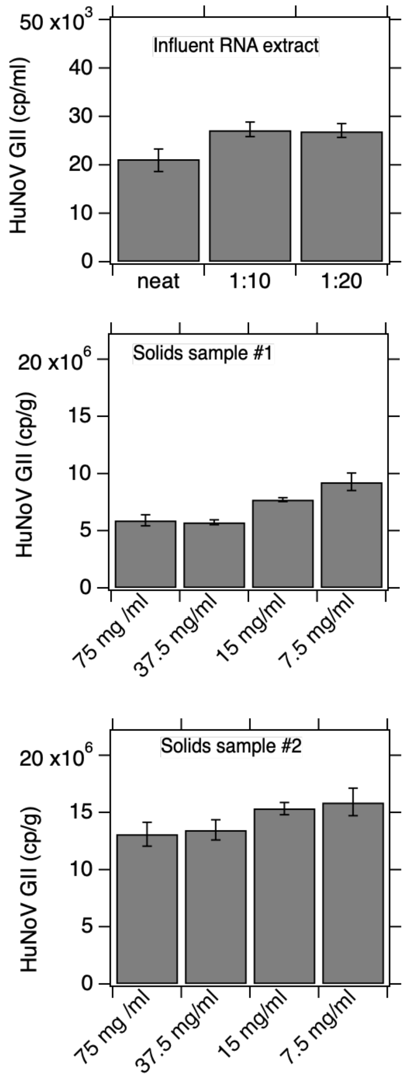

Figure S3. Data from inhibition testing. Concentration of HuNoV GII RNA is shown on the y-axis in all panels. Top panel. Concentrations derived using different dilutions of RNA extract from wastewater influent samples as template in the dd-RT-PCR; neat indicates not diluted. Similar results (less than 2X differences)[2] across dilutions indicate lack of evidence of inhibition. Middle and bottom panels. Results for concentrations in wastewater solids obtained using different mass concentrations of solids in DNA/RNA shield. Similar concentrations (less than 2X differences)[2] as the concentration is decreased suggests absence of inhibition.

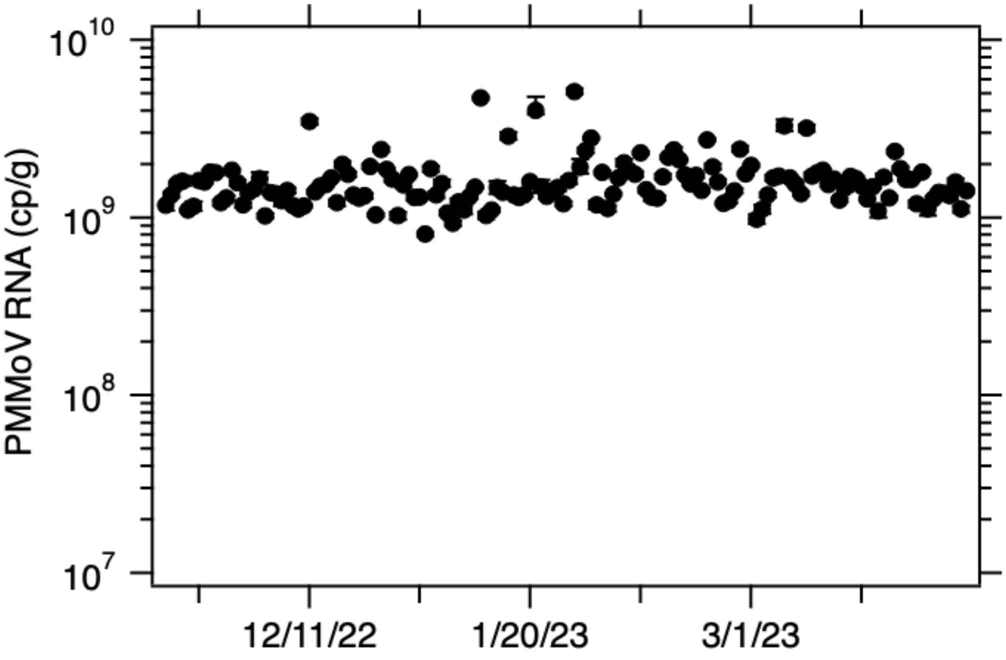

Figure S4. Concentrations of PMMoV RNA in wastewater solids from SJ WWTP. Errors are standard deviations. If the error bar cannot be seen, then it is smaller than the marker.

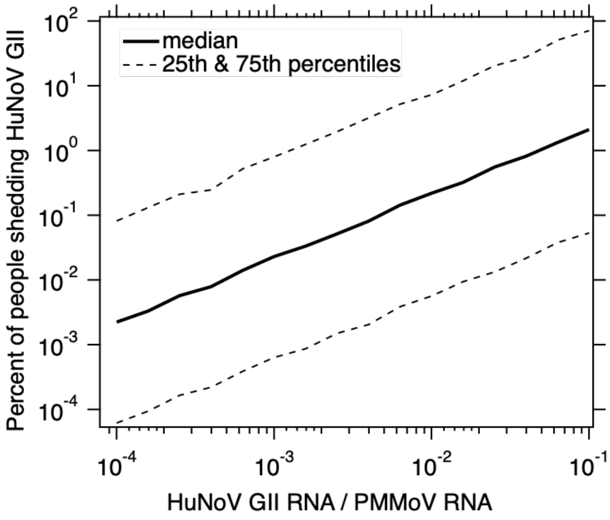

Figure S5. Modeled percent of individuals shedding HuNoV GII in their feces (F_{shed}) as a function of the ratio of HuNoV GII RNA and PMMoV RNA in wastewater solids.

Table S1: Details of the 145 WWTPs included in the study. The state where the WWTP is located, additional details regarding plant location, the sample type provided, the sample county and the reported population served. If an * appears next to the name (8 WWTPs total in CA), then 10 replicates were used to measure HuNoV, PMMoV and BCov. Otherwise, 6 replicates were used to measure HuNoV and 2 to measure PMMoV and BCoV.

**State**	**Plant**	**Sample Type**	**Sample Count**	**Population Served**
Alabama	Bessemer, AL	Liquids	50	225,000
	Cahaba River, Birmingham, AL	Liquids	34	95,000
	Fultondale, AL	Liquids	26	77,000
	Pinson, AL	Liquids	29	30,000
	Village Creek, Birmingham, AL	Liquids	50	200,000
California	Coastal, Laguna Niguel, CA	Liquids	47	48,000
	Contra Costa County, CA	Solids	42	484,800
	Davis, CA	Solids	88	68,000
	Esparto, CA	Liquids	46	4,006
	Fairfield, CA	Solids	50	155,000
	Fremont, CA	Liquids	31	229,476
	Gilroy, CA*	Solids (Settling w Imhoof cones onsite)	145	110,338
	Half Moon Bay, CA	Liquids	38	28,000
	Hollister, CA	Liquids	47	42,000
	Indio, CA	Solids	50	91,765
	JB Latham, Laguna Niguel, CA	Liquids	47	120,000

State	Plant	Sample Type	Sample Count	Population Served
	Lancaster, CA	Liquids	49	200,000
	Las Gallinas, San Rafael, CA	Liquids	50	30,000
	Lompoc, CA	Liquids	50	69,290
	Los Angeles County, CA	Liquids	51	3,500,000
	Los Angeles, CA	Liquids	51	4,000,000
	Los Banos, CA	Liquids	52	42,000
	Madera, CA	Solids	15	67,944
	Marina, CA	Liquids	44	262,000
	Merced, CA	Solids (Settling w Imhoof cones onsite)	50	91,000
	Mill Valley, CA	Solids	51	30,000
	Modesto, CA	Solids	50	230,000
	Napa, CA	Solids	46	83,300
	Newark, CA	Liquids	32	47,229
	Novato, CA	Liquids	50	53,000
	Oakland, CA	Solids	50	740,000
	Oceanside, San Francisco, CA*	Solids	140	250,000
	Ontario, CA	Solids	47	890,000
	Pacifica, CA	Liquids	49	40,000
	Palo Alto, CA*	Solids	145	236,000
	Paso Robles, CA	Solids	49	31,037

State	Plant	Sample Type	Sample Count	Population Served
	Petaluma, CA	Liquids	50	65,000
	Redwood City, CA*	Solids	145	199,000
	Regional, Laguna Niguel, CA	Liquids	46	129,000
	Riverside, CA	Liquids	32	350,000
	Sacramento, CA*	Solids	145	1,480,000
	San Diego, CA	Liquids	49	2,200,000
	San Jose, CA*	Solids	145	1,500,000
	San Leandro, CA	Liquids	50	50,000
	San Mateo, CA	Solids	46	150,000
	San Rafael, CA	Liquids	30	104,250
	Santa Cruz County, CA	Solids (Settling w Imhoof cones onsite)	49	160,000
	Santa Cruz, CA	Solids (Settling w Imhoof cones onsite)	49	160,000
	Santa Rosa, CA	Solids (Settling w Imhoof cones onsite)	47	230,000
	Sausalito, CA	Liquids	35	18,000
	South San Diego, CA	Solids	36	1,600,000
	Southeast San Francisco, CA*	Solids	142	750,000
	Sunnyvale, CA*	Solids	144	153,000
	Turlock, CA	Liquids	50	86,000
	Union City, CA	Liquids	31	68,150

State	Plant	Sample Type	Sample Count	Population Served
	Vallejo, CA	Liquids	48	121,000
	West Contra Costa County, CA	Liquids	43	100,000
	West Railroad, San Rafael, CA	Liquids	31	25,000
	Windsor, CA	Liquids	43	28,000
	Winters, CA	Liquids	46	7,286
	Woodland, CA	Liquids	50	59,000
Colorado	North, Parker, CO	Liquids	47	35,000
	South, Parker, CO	Liquids	48	25,000
Delaware	Seaford, DE	Solids	26	13,172
Florida	Altamonte Springs, FL	Liquids	44	95,000
	Eastern, Orange County, FL	Solids (Settling w Imhoof cones onsite)	45	195,299
	Jupiter, FL	Liquids	50	90,000
	Key Biscayne, FL	Liquids	28	829,725
	North Miami, FL	Liquids	35	776,150
	Northwest, Orange County, FL	Solids (Settling w Imhoof cones onsite)	47	66,690
	South Miami, FL	Liquids	35	920,528
	South, Orange County, FL	Solids (Settling w Imhoof cones onsite)	45	183,009
	Southwest, Orange County, FL	Liquids	44	50,000
Georgia	Big Creek, Roswell, GA	Liquids	50	189,593
	College Park, GA	Liquids	50	73,821

State	Plant	Sample Type	Sample Count	Population Served
	Columbus, GA	Solids	33	278,000
	Johns Creek, Roswell, GA	Liquids	50	84,486
	Little River, Roswell, GA	Liquids	50	12,818
	RM Clayton, Atlanta, GA	Liquids	48	94,000
	South River, Atlanta, GA	Liquids	48	94,000
	Utoy Creek, Atlanta, GA	Liquids	48	94,000
Idaho	Coeur d'Alene, ID	Solids	48	50,540
	Lander Street, Boise, ID	Liquids	36	108,556
	West Boise, ID	Liquids	36	186,901
Illinois	Glen Ellyn, IL	Solids (Settling w Imhoof cones onsite)	42	86,000
	Wheaton, IL	Solids (Settling w Imhoof cones onsite)	50	63,000
Indiana	Bloomington, IN	Liquids	44	56,090
	Downtown, Jeffersonville, IN	Liquids	46	25,000
	North, Jeffersonville, IN	Liquids	45	25,000
	South Bend, IN	Liquids	38	130,000
Iowa	Clinton, IA	Solids	36	29,300
	Coralville, IA	Liquids	31	23,000
	Marshalltown, IA	Liquids	33	27,400
	Muscatine, IA	Solids	50	24,400
	Ottumwa, IA	Liquids	49	25,529

State	Plant	Sample Type	Sample Count	Population Served
Kansas	Kaw Point, Kansas City, KS	Solids (Settling w Imhoof cones onsite)	39	90,000
	Lawrence, KS	Solids (Settling w Imhoof cones onsite)	44	80,000
	P20, Kansas City, KS	Solids (Settling w Imhoof cones onsite)	39	35,000
	Salina, KS	Solids	50	47,000
	Wolcott, Kansas City, KS	Liquids	38	15,000
Kentucky	Louisville, KY	Solids (Settling w Imhoof cones onsite)	14	423,913
Maine	Brunswick, ME	Liquids	39	10,000
	Portland, ME	Liquids	36	65,000
	York, ME	Liquids	32	10,000
Maryland	Hagerstown, MD	Liquids	45	90,000
	Hollywood, MD	Liquids	33	55,000
Massachusetts	Boston, MA	Solids	48	2,400,000
	Millbury, MA	Liquids	18	250,000
Michigan	Ann Arbor, MI	Liquids	50	125,000
	Jackson, MI	Solids	51	90,000
	Jenison, MI	Solids	51	75,000
	Traverse City, MI	Liquids	33	30,623
	Warren, MI	Liquids	46	140,000
Minnesota	Mankato, MN	Solids (Settling w Imhoof cones onsite)	48	70,000
	Rochester, MN	Solids (Settling w Imhoof cones onsite)	49	120,000

State	Plant	Sample Type	Sample Count	Population Served
New Hampshire	Dover, NH	Liquids	41	30,000
	Hall Street, Concord, NH	Liquids	49	45,000
	Penacook, Concord, NH	Liquids	48	4,000
New Jersey	Belmar, NJ	Solids	50	52,672
	Bridgeton, NJ	Liquids	11	50,000
	Newark, NJ	Solids	43	1,500,000
North Carolina	Kinston, NC	Liquids	49	25,000
	Winston-Salem, NC	Liquids	42	92,000
Ohio	Akron, OH	Solids	35	365,000
	Youngstown, OH	Solids	47	174,000
Pennsylvania	Chester, PA	Liquids	44	220,000
	Harrisburg, PA	Liquids	51	125,000
Texas	Gainesville, TX	Liquids	39	17,300
	Garland, TX	Solids	48	200,000
	Hollywood Road, Amarillo, TX	Liquids	48	60,000
	River Road, Amarillo, TX	Liquids	43	140,000
	South, Laredo, TX	Liquids	42	120,000
	Sunnyvale, TX	Solids	48	186,000
	Wichita Falls, TX	Solids	49	90,000
	Woodlands SJRA WWTF No. 1, TX	Liquids	21	65,000
	Woodlands SJRA WWTF No. 2, TX	Liquids	21	70,000

State	Plant	Sample Type	Sample Count	Population Served
	Woodlands SJRA WWTF No. 3, TX	Liquids	21	15,000
	Zacate Creek, Laredo, TX	Liquids	41	140,000
Utah	Central Salt Lake Valley, UT	Solids	50	600,000
	Provo, UT	Solids (Settling w Imhoof cones onsite)	33	115,000
Vermont	Essex Junction, VT	Solids	16	30,000
	Montpelier, VT	Solids	15	10,100
Virginia	Aquia, Stafford, VA	Solids	17	100,000
	Hillsville, VA	Solids	49	3,000
	Little Falls Run, Stafford, VA	Solids	16	50,000
